# Stimulation of Rabbit Squamous Epidermis Cells Using Extracts of Mistletoe *Dendrophthoe pentandra* L. Miq in a Topical Gel

**DOI:** 10.1155/vmi/4081052

**Published:** 2025-05-25

**Authors:** Lazuardi Mochamad, Chi-Hsien Chien, Jie-Long He, Eka Pramyrtha Hestianah, Hani Plumeriastuti

**Affiliations:** ^1^Sub-Division Veterinary Pharmacy Science, Faculty of Veterinary Medicine, Airlangga University, Mulyorejo Rd, “C” Campus Fakultas Kedokteran Hewan Universitas Airlangga, 3rd Floor Building, Surabaya 60115, Indonesia; ^2^Department of Post-Baccalaureate Veterinary Medicine, Asia University, Taichung 41354, Taiwan; ^3^Sub-Division Veterinary Histology, Department of Veterinary Medicine, Faculty of Veterinary Medicine, Universitas Airlangga, Mulyorejo Rd, “C” Campus Fakultas Kedokteran Hewan Universitas Airlangga, 2nd Floor Building, Surabaya 60115, Indonesia; ^4^Division of Veterinary Pathology, Department of Veterinary Medicine, Faculty of Veterinary Medicine, Universitas Airlangga, Mulyorejo Rd, “C” Campus Fakultas Kedokteran Hewan Universitas Airlangga, 1st Floor Building, Surabaya 60115, Indonesia

**Keywords:** epithelialization, genistein, good health, *in vivo* test, mistletoe, quercetin-like compound

## Abstract

Quercetin-like compounds (QLCs) are secondary metabolite compounds of flavonol found in the leaf extract from the mistletoe *Dendrophthoe pentandra* L. Miq. This study aims to determine the ability of QLC to stimulate epithelialization in rabbit skin. The leaves were macerated with methanol, ethyl acetate, and n-hexane solvents, and crude macerates were separated and purified into QLC using preparative high-performance liquid chromatography. The purified QLC as an analyte was prepared in serial concentrations of 4.5%, 6%, 8%, and 10% and then formulated by new methods as topical gel preparations. The epithelialization stimulation test was performed on 60 rabbits divided into 20 rabbits for the trial group, the other 20 rabbits for the positive control group, and negative control groups. The trial group was split into 4 trial subgroups for topical gel application at each concentration. Gels were topically applied to the exfoliated skin of rabbits in the trial group twice a day for 5 days. The probit analysis showed that an epithelialization of 25%, 50%, and 75% of the in vivo study endpoint was found at a QLC concentration of 4.644%, 5.185%, and 5.790%. Rectangular cuboidal QLC particles with an average size of 0.01 μm–0.1 μm have shown the ability to stimulate epidermal epithelial proliferation starting from a concentration of 4.5% in topical gels with new formulations that can boost the epithelialization of the upper layers of the skin (*p* < 0.05).

## 1. Introduction

Using herbal-based medicines supports the Sustainable Development Goals (SDGs) activities, especially point 3, namely, good health and well-being [1]. This is because the side effects of herbal compounds are relatively nonexistent, and drug chemical residues are not produced [2]. These advantages will be beneficial if the medicine for veterinarians will be preparations based on plants or, more specifically, in topical preparations [3]. Topical preparations are stated specifically considering three things that will have a direct impact on the safety of the use of veterinary drugs, namely, (a) safe for the drug, (b) safe for the environment, and (c) safe for humans who consume fresh products of animal origin [4, 5]. In cases of lymph skin diseases (LSDs) in ruminants and foot and mouth disease (FMD) infection in cattle with manifestations of blisters around the mouth and feet, it is very beneficial if the active ingredients used are derived from medicinal plants [6, 7]. Similarly, blisters around the udder nipples in dairy cows are due to milking, and topical infections in horses are due to myiasis cases [8, 9]. From the perspective of ethnopharmacology, it is known that many medicinal plants are already useful for topical therapeutic use both as antimicrobials and skin epithelial proliferation, which are often used for veterinary cosmetics. In the field of cosmetics as well as the area of skin treatment, it is known that not many medicinal plants have been developed for this purpose. More specifically, medicinal plants can stimulate the epithelialization process in skin wounds (epidermis and dermis).  Table 1 is one example of a medicinal plant that can stimulate skin epithelialization in animals.

The search for secondary metabolites of medicinal plants should, in principle, follow five main provisions: (a) using plants that are not rare or protected from extinction, (b) that thrive in more than two regions of countries or continents, (c) that can be cultivated intensively, (d) where the empirical efficacy of plants in the field has been determined, even if unexpectedly, and (e) excluding medicinal plants that are prohibited in many countries or excluding medicinal plants such as marijuana [14–16]. One type of medicinal plant that meets these five-point criteria is the mistletoe group of plants, which are parasitic plants that grow on the host plant. Mistletoe plants are diverse, and one type that grows on the *Lansium domesticum* parent plant is *Dendrophthoe pentandra* L. Miq., which is identified in the Southeast Asian-Australia region (Australia, Brunei Darussalam, Papua New Guinea, Malaysia, New Zealand, Philippines, Thailand, and Timor-Leste). The plant was grown well in Indonesia and identified as *Benalu Duku* (BD). In Taiwan, the BD plant is known as the mistletoe of a host plant called *Lengkong* and can thrive in tropical and subtropical regions. The initial study of this medicinal plant began in 2000 with an empirical history, namely, the use of BD leaves that have been processed via infusion or decoction process and are subsequently drunk by patients with breast cancer [17, 18]. BD has recently been reported to exhibit several benefits related to pharmacodynamic properties. These properties were determined after phytochemical extracts separated bioactive secondary metabolites. Quercetin-like compounds (QLCs) were separated from BD leaves and shown to be efficacious as androgenic, antiviral, and antidiabetic compounds [19–21]. The bioactivity of QLC in the BD plant can be attractive for using these as mosquito-repellent compounds [22]. The most important substance of these QLCs was the flavonoid elements, especially the flavonol component of the plant, which are polyphenol compounds plus a small content of genistein. The combination of these two bioactive molecules has been proposed to stimulate eukaryotic cell growth, and the two components can work mutually to stimulate the epithelialization of eukaryotic cells [23]. This is because genistein compounds contain complex aromatic elements that have a high bond energy that is difficult to substitute or add. This synergistic cooperation between these two components is predicted to stimulate the mutual replication of cellular DNA in cells through the lipophilic part of the lipid bilayer of epithelial cells and thereby accelerate cell multiplication [24, 25]. This induces epithelialized growth and is generally suitable for application to parts of the skin that experience exfoliation [26]. QLC was very dominant in promoting the stimulation of epithelial proliferation, while the most important elemental parts are quercetin-3-O-rhamnoside, quercetin-3-galactoside-7-glucoside, and robinetin. These compounds have been found to have significant biological activities as an epithelialization, including anti-inflammatory and wound-healing properties, which contain an aromatic complex rich in −NH− groups to stimulate metabolism cells cell eukaryotic type [27, 28]. Thus, these compounds are suitable for further development efforts for skin regeneration in cases such as burns, exfoliation of the skin due to physical injuries due to incisions in livestock, and accelerated regeneration for the cosmetic field of pets such as dogs and cats. The novelty is also necessary and useful for cases of a reemerging disease such as cutaneous herpes and other viral diseases such as contagious ecthyma and milker's nodules [29]. With epithelialization stimulation techniques, treatment with these compounds is expected to inhibit viral infection of eukaryotic cells [30, 31].

The most important principles for the stimulation of cell epithelialization are (a) the size of a bioactive molecule, (b) the attachment between the bioactive molecule and the target cell, and (c) the concentration of the bioactive molecule in the required area [31, 32]. These three points ensure the formula contains bioactive solutions directly prepared based on drug-carrier ingredients. The strategy has never been performed for research or veterinary therapeutic applications. The first principle regarding the size of bioactive particles implies that the smaller the drug particles, the larger the surface area of the drug that will have to attach to the clinical target. Furthermore, small-sized drug particles have high solubility in oil and water media, and in such conditions, the drug will more easily penetrate the lipid barrier in the body. Finally, metabolizing a smaller particle is easier [31]. The second principle is the attachment between the bioactive molecule and target cells, depending on the strength of the drug-target bond and the resistance of this bond to disruption. Theoretically, the type of chemical bond that is strongest and most resistant to disruption is the *Van Der Waals* bond, as well as new molecular forms that have both single and complex aromatic forms, which can balance the strength of ions between atoms [32]. The third principle is that the distribution of bioactive drugs must be homogeneous so that all parts of the target can be treated with the drug at the same concentration. The third principle requires a homogeneity value between 100% and 120%. The third principle is also often used for mixing drugs with feed for chickens (medicated feed) as well as the main requirements for unguentum dosage from drugs [33, 34]. These three principles are novel steps in developing topical drugs that were used as the basis for preparing animal medicine formulations in this research. These three principles are highly suitable in designing new bioactives based on the spirit contained in the healthy lifestyle in the SDGs and avoiding the possibility of antimicrobial resistance if such bioactives are developed as antibiotics. Therefore, this study evaluated the efficacy of QLC for epithelial stimulation in rabbit skin as an in vivo test, which was chosen because this model can quickly provide results. Rabbits are used because the effect of cell proliferation is easily observed in their epithelium as a reference to similar research analogies although different research objects [35, 36]. This study hypothesizes whether QLC extracted from BD leaves can increase the stimulation of epithelial cell proliferation in rabbit skin and what initial concentration can stimulate epithelial proliferation.

## 2. Materials and Methods

### 2.1. Research Design

**Figure 1:**
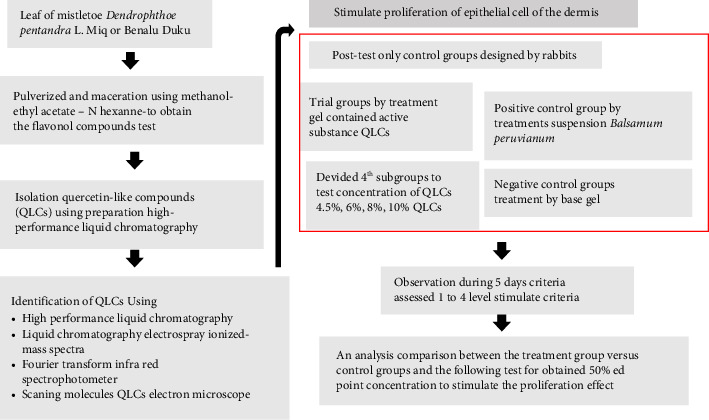
Research workflow stimulated epithelialization of quercetin-like compounds to dermis skin of rabbits.

The research design was a posttreatment design with trial, positive, and negative control groups. Each research group required several experimental animals divided into four subgroups, according to the concentration of analytes that were tested. The analytes as identified by QLC were obtained from the extract of organic solvent described below from the leaves of a plant of mistletoe, namely, *Dendrophthoe pentandra* L. Miq that grew up well in *Lansium domesticum* in the area Muara Enim district South of Sumatera—Indonesia (Latitude −4.2327° or 4° 13′ 58″ south, and Longitude 103.6141° or 103° 36′ 51″ east). Animals in the trial subgroup were administered with a topical gel containing one of four concentrations (4.5%, 6%, 8%, and 10%). The concentration between 4.5% and 10% was suitable for topical gel preparations and had a water mass balanced with the fat/oil mass. A range of 4.5%–10% has been set for patent registration. The reason is that the lower limit of 4.5% is the result of a preliminary study. Meanwhile, the upper limit of 10% is done with the consideration that the gel capacity for a wound of 3 × 3 cm^2^ requires 65 G. Meanwhile, QLC should be in a complete dissolution atmosphere with dimethyl sulfoxide (DMSO) in a ratio of 1:50. To maintain the balance of gel cohesiveness, the oil fraction can be stabilized with carbomer elements. If the addition is above 10%, more solvent will be added than the 1:50 fraction. This will make the gel formulation unstable. The control subgroup (positive control and negative control) was applied as other composition of gel. The research flow diagram is shown in Figure 1. This study also designed a new topical gel formula that can adhere to the skin of experimental animals for a long period but does not cause irritation.

This research work was conducted from July 2023 to March 2024. The BD leaves were collected from 2021 to 2022 because the appearance of BD occurs in the rainy season between November and January of the following year. The taxonomic identification referred to the Biology Plant Department Indonesia Research Authority of Plants no. Letter B-1679/11.6.2/D1.05.07/6/2023, date 7^th^ of June, 2023. The research was performed at Airlangga University and Asia University in Taichung, Taiwan, more specifically, Campus C, Airlangga University, namely, the Faculty of Veterinary Medicine, Airlangga University, and the Department of Post-Baccalaureate, Veterinary Medicine, Asia University-Taiwan. The clarification used for experimental animals was controlled and assessed through the Animal Care and Use Committee (ACUC), Faculty of Veterinary Medicine, Universitas Airlangga, Mulyorejo Rd. “C” Campus Surabaya—Indonesia. Approval for using experimental animals with a certificate of completion of the ethical testing of experimental animals was obtained with authorization number 1.KEH.019.02.2024. The certificate of animal ethics clearance received approval from the testing team on behalf of the Dean of the Faculty of Veterinary Medicine, Universitas Airlangga, on February 5, 2024.

Animal experiments determined an Indonesian local rabbit (family *Leporidae*, genus *Oryctolagus*, and species *Oryctolagus cuniculus* with strain angora) at criteria inclusive to be a healthy adult male weighing an average of 2.7–3 kg. Rabbit exclusive criteria were defined when excluding inclusive criteria. Rabbits were obtained from a specialized research breeding center with complete health control by a local veterinarian at the Indonesian Center for Veterinary Pharmaceutical Analysis and Application at the Subdivision of Veterinary Pharmaceutical Sciences, Faculty of Veterinary Medicine, Airlangga University, Surabaya, Indonesia.

Rabbits before the experiment were adapted for 7 days in the experimental animal cage of the Faculty of Veterinary Medicine, Universitas Airlangga. Adaptation for 7 days is done by checking the health of rabbits by measuring temperature and body weight. The condition of the cage is determined by the Faculty of Veterinary Medicine, Universitas Airlangga, Section of the Animal Experimental Unit, with a room width of 3 m, a room length of 5 m, a room height of 3 m, and equipped with air conditioning and room temperature. Room humidity is set at 25% and room temperature is set at 20°C. After adaptation, the rabbits were separated between treatment and control rabbits, followed by treatment.

### 2.2. The Sample Size for Trials

The calculation for the number of experimental animals needed is based on the principle that each drug concentration will be tested on a subgroup of experimental animals. Considering that the QLC concentrations tested comprise four concentrations, the animal requirements for each concentration were calculated using the formula below (*N*):

(1) $$\begin{array}{c} N=\frac{{\left[\left(Z_{1}-\left(\alpha /2\right)\right)+Z_{\beta }\right]}^{2}}{\left({\left(d\right)}^{2}/\left({\left(\text{Sa}\right)}^{2}+{\left(\text{Sb}\right)}^{2}\right)\right)}. \end{array}$$

Equation (1) with a significance of 0.05 at the 5% margin of error with the following criteria; Z_1_ − (α/2) = 1.96;  Z_β_ = 1.645; d = 3.62;  S_a_ = 1.7;  S_b_ = 1.4. Zβ was determined based on populations at equal samples [37]. Sa and Sb were calculated as two populations with equal samples [38]. Formula (1) requires *N* = 4.88 animals, which was rounded up to five animals. Thus, each test concentration required five experimental animals in each subgroup.

### 2.3. Extraction of *Dendrophthoe pentandra* L. Miq (BD) Leaves to Obtain QLC

#### 2.3.1. Isolation and Identification QLC

The QLC separation technique was carried out using a mobile maceration technique at a room temperature of 20°C–22°C for 1–3 days, depending on the acquisition of raffinate. A total of 3 kg of BD leaf powder was subjected to mobile maceration with the following steps: every 500 g of BD powder was added to 1 L of polar solvent as methanol (pro analysis grade, Merck, Darmstadt, Germany, catalog number 106009), and then shaking was performed for 3 days via rotary displacement. The extract was filtered using a porcelain Buchner funnel (Fisher Scientific catalog number FB 966J, Pittsburgh, US), and then the macerated substances were squeezed out using a flannel cloth. The filtrate was dried using nitrogen vapor in a water bath temperature set to 40°C (Precision Circulating Water Baths Fisher Scientific , Pittsburgh, US). The macerate from the extract methanol process was obtained at 900 g of crude extract BD. The thickened 600 g macerate was added to ethyl acetate solvent as a semipolar solvent (Merck, Darmstadt, Germany, catalog number 109623) in a split tube with a 100 g macerate to 250 mL ethyl acetate (ratio 1:2.5). The ethyl acetate solution was then collected and dried using nitrogen vapor in a 40°C water bath. Furthermore, the macerate from the dried ethyl acetate at 120 g was then macerated in a separate flask using normal hexane as a nonpolar solvent (Merck, Darmstadt, Germany, catalog number 104374) and water for chromatography (Merck, Darmstadt, Germany, catalog number 115333) at a 50:50 ratio. The resulting macerate at 120 g was dried using a rotary evaporator at 40°C (Type R-220 production Buchi Corp., Switzerland), and the final macerated viscous compound containing QLC was used in a flavonoid test [39]. The flavonoid content test was performed via the Willstatter method, where 1 mL of macerated polyphenol sample was added with magnesium chloride powder (Merck, Darmstadt, Germany, catalog number 208337-100G) and two drops of concentrated hydrochloride acid (HCL) pro analysis grade (Merck, Darmstadt, Germany, catalog number 109060). Shaking was performed until well mixed. The polyphenols will undergo a hydrolysis reaction with magnesium chloride powder in HCL and methanol to produce benzopyrylium or flavylium salts that have a dark red color [21]. QLC was isolated using preparative high-performance liquid chromatography (HPLC) with quercetin standard [22]. An Agilent Infinity II 1260 with Diode Array Detector (DAD) System was used for preparative HPLC (California, US) at a flow rate of 0.01–50 mL/min, operating pressure of 420 bar/6092 psi, pH range of 1.0–12.5, isocratic formation by high-pressure binary mixing, flow accuracy of < ± 1.0%, composition accuracy of ± 1.0%, flow precision of ≤ 0.3, and composition precision of ≤ 0.3% relative standard deviation. The HPLC system tool kit 1260 Infinity II specifications were as follows: an injection range of 0.1–900 μL up to 3600 μL, injection precision of 1 μL at < 5%, 5 μL at < 2%, 10–50 μL at < 1%, and the injection volume 500 μL–3600 μL at < 0.25%, pressure range 0–400 bar, 0–58,001.51 Psi, sample viscosity range of 0.2–5 cp, sample capacity of 132 × 2 mL vial, carryover of < 0.05%, and injection cycle time for less than 60 s for an injection volume of 900 μL. A reversed-phase preparative column (Agilent, California, US, catalog number 446905-302; 100 Å C_18_, 30 × 50 mm, 5 μm) was used. The mobile phase was water pro-HPLC (Merck, Darmstadt, Germany, catalog number L0015-BC): methanol pro-HPLC (Merck, Darmstadt Germany, catalog number 106009) at fraction 40:60 and a flow rate of 2.5 mL/min with detection at 250, 370, 375.4, and (reference) 360 nm with a stop time of 30 min. The autosampler collectors were determined at 21–23 min after injection of 50–75 μL of QLC onto the column and then compared with standard quercetin at certified reference material (CRM) from Sigma Corp. (Darmstadt, Germany, catalog number Q4951-10G). Isolated QLCs were dried using nitrogen gas (PT Samator, Jakarta-Indonesia) in a water bath at 40°C (Memmert water bath WNB 22, Mi, US). Identification analysis was performed using a preparative HPLC system with a preparative and analytical column as follows: C_18_ column (30 × 250 mm, particle size 5 μm, 400 bar, S/N 44905-303, Agilent, California US) and C_18_ column (10 × 250 mm, particle size 5 μm, 400 bar, S/N 440905-802, Agilent, California US). The detection wavelengths were 250, 370, 375.4, and (reference) 360 nm with a stop time of 30 min. The flow rate was adjusted at 0.5–1 mL/min with a 5 μL injection into the column. The mobile phase was water:methanol at a 40:60 ratio at approximately 20°C−22°C. The laboratory room of HPLC was adjusted at 20°C, 56% humidity.

The liquid chromatography-electrospray ionized-mass spectra (LC-ESI-MS) were obtained using ultraperformance liquid chromatography-mass spectrometry (UPLC-MS) controlled by the UNIFI software program (Waters Corp., Frankfurt, Germany) and using a C_18_ column (Agilent, California, US, catalog number 5982-1111). The oven of the cage of the column and autosampler injectors was set to 20°C–22°C, while the injector volume capacity was set to 10–15 μL. The running application set gradient models using the mobile phase A acetonitrile (Merck, Darmstadt, Germany, catalog number 100003) containing 0.1% formic acid (Merck, Darmstadt, Germany, catalog number 801081) and mobile phase B (chromatography water containing 0.1% formic acid). The flow rate was adjusted to 0.5 mL/min. The MS parameters were adjusted to the time-of-flight (TOF) MS E mode using ESI at ± and an acquisition range of 50–1200 Da. The following analysis criteria were adopted: mass error reading analyte of ≤ 5 ppm, isotope match m/z root mean square (RMS) of ≤ 5 ppm, isotope match m/z RMS% of ≤ 8 to 10%, and an analyte intensity of ≥ 250 to 300. For one fraction, the brake value was < 3-4 for the fragment elucidation system.

For the identification of the QLC, the results were compared with those of the quercetin standard using a Fourier-transform infrared spectrophotometer (FT-IR). FT-IR was performed using a PerkinElmer Spectrum One (Shelton, CT 06484-4794, US, Models PN 09934358 by the KBr-Transmission) with a parameter range of 4000–400 cm^−1^, duration scan of 20 s, and resolution of 4 cm^−1^. The sample tablets were prepared using the following devices and materials: agate mortar and pestle, stainless spatula, sample holder (holder press tablet consists of a barrel and 2 bolts), 3/4 size wrench, and adjustable wrench size at 12 inches, KBr FT-IR grade. The tablet-making technique was carried out by inserting sufficient KBr powder into the holder, adding a sample in a certain quantity, and adding KBr powder in the same amount as the first the holder is pressed as hard as possible and screwed for 10 min so that a KBr tablet is formed. Interpretation of the activities of vibration atoms was assessed using IR spectrograms, especially in the diagnostic area and fingerprint area by referring to https://spectrabase.com/ and https://webbook.nist.gov/chemistry/name-ser/, https://chem.libretexts.org/Ancillary_Materials/Reference/Reference_Tables/Spectroscopic_Reference_Tables/Infrared_Spectroscopy_Absorption_Table.

Furthermore, the morphological structure of crystals of QLC and those of standard quercetin molecules was evaluated using a Phenom ProX Desktop scanning electron microscope (SEM) with energy-dispersive X-ray diffraction (EDS). The SEM was adjusted as follows: electron high tension (HET) at approximately 15,000 kv, working distance of approximately 5–10 nm, magnification of 160–3,500,00 ×, and scale of the object of approximately ≤ 8 nm. The object of SEM examination is dry and stable particles at a certain level of thermal energy. The calculation of the surface area of QLC particles was carried out using ImageJ software (https://imagej.nih.gov/ij/index.html accessed on May 5, 2024). Analysis of the surface area size of particles using OriginPro 2018 software (Production of Origin-Lab Corporation, One Roundhouse Plaza, Suite 303, Northampton, MA 01060, USA).

#### 2.3.2. Standardization of Isolate QLC

QLC standardization was carried out in 4^th^ method in tests that are commonly carried out as follows.

##### 2.3.2.1. The 1^st^ Method for Assessing Flavonoid Content Test

QLC extract 0.5 mg was dissolved in 10 mL of methanol (p.a), the solution formed was added with 0.5 mL of 1% (w/v) ferric chloride (III) solution, Merck catalog number 7705-08-0. Shake the mixture well and then observe to determine whether the flavonol compound is causing a bluish color change. Second method: 1 mg of raffinates dried of QLC was dissolved in 10 mL of methanol p.a (w/v). Place the 2 mL of solution QLC in three tubes, followed by tube A (control tube), tube B control indicator, and tube C as a test sample. A prepared 0.01 N hydrochloric acid (Merck catalog number 109057) was dissolved in aqua demineralized, 50 μL of the resulting solution was taken and dripped into tube B and tube C. Tube B was placed onto the hot plate for 5 min, and then the hot plate device was set at 100°C until the solution became concentrated. Tube C adds the 1 mg of Zinc p.a (Merck catalog number 7440-66-6). The C tube was left to stand for 5 min, and the color change was observed, compared to tubes B and A. Tube C appeared green as indicated, containing the flavonoid. Tube A was brown, and tube B was available in a dark color [21, 22].

##### 2.3.2.2. The 2^nd^ Method Evaluates the Retention Factor (*R*_*f*_)

The TLC containing activated magnesium silicate was prepared (TLC product from Supelco Corp., catalog number 1343-88-0). The analyte isolate QLC was dissolved by methanol and adjusted to 5 ppm. The standard of QLC at 5 ppm was prepared by dissolving in methanol. Next, QLC and standard quercetin were dropped on TLC paper at 5 μL volume, then elution was carried out using the 1-butanol p.a (Merck catalog number 101990):acetic acid p.a (Merck catalog number 64-9-7):pure water free-mineral (Product PT Kimia Farma, Indonesia) at 1:1:1 mobile phase solution. Spray the TLC using a solution of AlCl_3_ by Sigma-Aldrich Corp., US catalog number 563919, at 10% in pure water-free mineral (w/v). The TLC paper was dried using warmer devices at 37°C for 15 min. The stain was measured *R*_*f*_ at wavelengths of 254 nm and 365 nm using a TLC viewing chamber model UvOc-O2 (Lab Store-Indonesia) [21, 22].

##### 2.3.2.3. The 3^rd^ Method Re-Evaluates the Physicochemical Characterization of QLC Analytes and Specific Chemical Bonds in Infrared Spectra Using HPLC and FT-IR

The standardization of QLC isolation results was carried out by conducting a reverse test to determine the stability of the isolate through a preparative HPLC device with the same system as at the beginning of isolation. The quality of similarity was reviewed from the chromatogram area and retention time on the same HPLC system, along with the type of column and fraction of the mobile phase eluent fraction. The HPLC used Agilent Infinity II 1260 type with detector kind of Photodiode Array. The method was used by isocratic with eluent mobile phase methanol:water at fraction 40:60. The wavelength was set at 250, 370, 375.4 nm, and (reference) 360 nm at a flow rate of 0.5 mL/minute, and column C_18_ was used (10 × 250 mm, particle size 5 μm, 400 bar, S/N 440,905-802, Agilent). The volume injection was used at 50 μL and set for a stop-time of 30 min. Standardizing the results of QLC sample isolates, a similarity test was carried out using FT-IR as done in the subchapter Methodology, Isolation, and Identification. Furthermore, the spectrogram obtained is overlaid in the area of the fingerprint wavenumber to determine the level of similarity of compounds compared to the quercetin standard.

##### 2.3.2.4. The 4^th^ Method for Re-Evaluating Total Quercetin at Isolates QLC

Test the quercetin content in the analyte by adding aluminum chloride using a UV spectrophotometer. The principles were as follows: The purity isolate of the QLC sample contains keto and hydroxyl groups. The keto and hydroxyl structures bound to aromatic compounds will react with aluminum chloride to produce a yellow indicator color. The color indicator can be observed at visible wavelengths at 370 nm to 505 nm. The method used is quantitative analysis using commercial quercetin standards. A sample of 10 mg was dissolved in 10 mL of methanol as the analyte. Take 1 mL of analyte and add 0.2 mL of 10% (w/v) AlCl3 solution and 0.2 mL of 1M potassium acetate (Sigma-Aldrich corp., catalog number P5708-500G) both in demineralized water. The last step is to add 10 mL of demineralized water and let it stand at room temperature for 30 minutes. Absorbance is measured at a wavelength of 370 nm to 505 nm from a UV-visible spectrophotometer.

### 2.4. Preparation of New Topical Gel Formulation Containing QLC

The topical gels were prepared at two stages, first for the base of gel and the second as a mixed base gel and active substances. The first stage was to create a stable and gentle gelling formula with an acid-base balance set between 6.5 and a maximum of 6.8. Carbomer 940 (Spectrum Chemical MFG Corp., NJ, US catalog number NF CAS 9003-01-4) 1.6% (w/v) was added to 65 mL of demineralized water (PT. Brataco-Jakarta, Indonesia) and then mixed and stirred for 35 min gradually followed by homogenization at 90–140 rate per min (rpm) for 20 min (Homogenizer GLH 850, Product Omni International, Georgia, US). The carbomer and water were added to triethanolamine as a mild preservative (Merck, Darmstadt, Germany, catalog number 108372) 0.65 mg, followed by sodium ethylenediaminetetraacetic acid (Na-EDTA) as a stabilator substance from Merck, Darmstadt, Germany, catalog number 108454. A 1% dissolved solution used 20 mL of demineralized water with 15 g of propylene glycol liquid (Merck, Darmstadt, Germany, catalog number 817003). The mixed compounds of Na-EDTA and propylene glycol were homogenized. The second stage: QLC compounds were prepared at 4.5%, 6%, 8%, and 10% (w/v) by using 2.925, 3.90, 5.20, and 6.50 g of QLC macerate, respectively, followed by ultrasonication to improve dispersion in the topical gels and then by mixing with the mixed compound Na-EDTA and propylene glycol and stirring for 30 min. Compounds 1, 2, and 3 were mixed, and the viscosity was measured using a digital viscometer NDJ-8S rotor No. 3 at a dynamic viscosity of 2 MPa^.^s (Shanghai Lichen Bangxi Instrument Technology Co., China) after homogenization at 30 min. The formulation of topical gels using QLC at 4.5%, 6.0%, 8.0%, and 10.0% (w/v) is shown in Table 2. The composition and mixture to produce a new topical gel formulation were referred to at https://patentscope.wipo.int/search/en/detail.jsf?docId=ID434619094.

The quality control of the gel was workflow at three stages: the homogenizer test, the stability test, and the stickability test on a 60-degree slope. The homogeneous test was conducted by placing 1 g of gel at the center point of a 5 cm diameter circle on a transparent glass slide. Furthermore, press using transparent glass against the mound of gel until the mound becomes thin and flat and observe the results of pressing. If the outer circle of the gel pressing results in a circle that is similar to the circle made, it is declared homogeneous. Stability tests were conducted by testing the stability of up to fourteen test cycles. Cycle 1: place the gel at 40°C for 24 h in an incubator and continue at 4°C for 24 h in a refrigerator. This method was repeated up to fourteen times each day. Observations were made of the gel preparation; if the phenomenon of syneresis appeared, the gel made was unstable. The adhesion test is carried out by applying to an artificial skin object that has been marked with a 5 cm diameter circle where the artificial skin object is placed with a slope of up to 60 degrees and observed. If the gel dissolves in less than 60 min, it means that the adhesion of the gel is not strong enough [40–42]. The novelty of the gel's new findings is that it contains mistletoe extract that is only soluble in DMSO, while it is known that gels contain large macromolecular elements that are penetrated by small molecules. Thus, for the DMSO soluble extract, if no known water element can form a gel, the stability of the gel is damaged.

### 2.5. Analysis of Epithelial Skin Proliferation

Trial rabbits were subgrouped into four groups with five rabbits per subgroup as in equation (1). Subgroups 1 to 4 of the trial group contained five rabbits per concentration as a replicate of the target research. The subgroups of positive control and negative control were adjusted at 20 rabbits. At the beginning of the rabbit experiment, a 3 × 3 cm^2^ area on the rabbit's back was cut until there was no fur, and then 1% (w/v) betadine solution was applied to the area. A solution of 0.1% (v/v) procaine HCl as a local anesthesia solution was then injected 0.1 mL into the subcutaneous. Five min after injection, the outer skin was scoured using scalpel number 12 and immediately covered with sterile gauze. The result of the wounding is immediately measured, starting from the upper limit of the skin scraping result (A) to the lower limit of the skin scraping result (B) using distance-measuring equipment (mm). Topical gel smearing was performed at 4.5%, 6%, 8%, and 10% (v/v) of QLC. Gel application was performed twice daily using topical gels according to the percentage tested by each group, then as soon as possible to evaluate epithelial proliferation. After the gel application, the wound was covered with sterile gauze. Treatment lasted up to 5 days. To examine the proliferation of rabbit skin cells, a magnifying device was used at 1700 × for 0.5 min with a light-emitting diode lamp (type 3017, GIMA Corp., Italy). Observations were made by observing the wounded skin with scoring using four levels (1–4). The direction of observation in one field of view follows the twist of the letter S, starting from the dorsal part of the observation area and ending in the ventral part of the observation area. The observation was repeated up to three times. Changes from the observations of each rabbit were recorded and photographed to re-evaluate the changes, including any detrimental to the rabbit's well-being. The criteria for the examination of epithelialization were as follows:

  Level 1: In the scraped wound, bleeding *petechiae* were present, the limbus in the wound area was swollen, part of the endodermis was present, and the peripheral part around the scraping in the epidermis appeared red. When pressed, the rabbit felt pain and avoided this. The size of the upper limit of epithelial scrapings (A) to the lower limit of epithelial scrapings (B) was between 75% and 100% of the first size of epithelial scrapings.
  Level 2: No *petechiae* were found in the wound, but the limbus of the scraping wound area is rounded. The color of the peripheral part of the epidermis has started to normalize, and when pressure was applied to the scraped area, the rabbit no longer made avoidance movements, a sign that it did not feel pain. The size of the upper limit of epithelial scrapings (A) to the lower limit of epithelial scrapings (B) was between 50% and 75% of the first size of epithelial scrapings.
  Level 3: New skin had begun to grow in the wound area, and the wound limbus had disappeared; pain had disappeared. The size of the upper limit of epithelial scrapings (A) to the lower limit of epithelial scrapings (B) was between 25% and 50% of the first size of epithelial scrapings.
  Level 4: Epithelialization of the skin has occurred, and the limbus of the scraped wound has disappeared; old epithelial cells may have dried out, and a degree of hair growth was present. The size of the upper limit of epithelial scrapings (A) to the lower limit of epithelial scrapings (B) was between 0% and 25% of the first size of epithelial scrapings.

**Figure 2:**
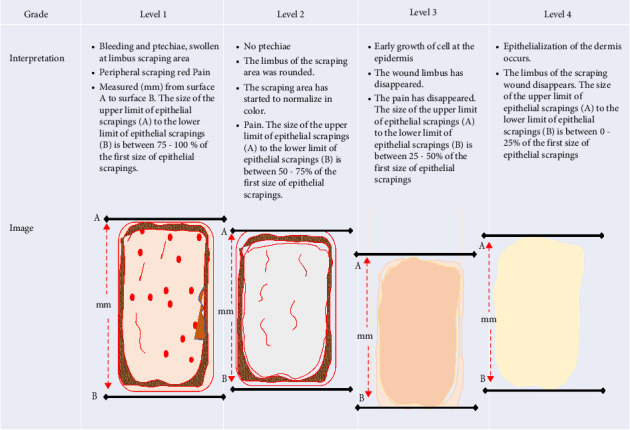
Criteria for assigning the level of epithelialization in the cell epidermis of rabbits.

Furthermore, levels 1 to 4 of the process affected the stimulation of epithelial proliferation on the epidermis of rabbit skin as shown in the shaded image in Figure 2. The wound reduction process was measured using a scaling rule (mm) starting from the outermost wound circle. These criteria were prepared in several previous reports and are specific to rabbit skin as an in vivo test [43, 44]. Treatment for the negative control group was to exfoliate the epidermis skin using a gel base as described in preparations of a new topical gel formulation containing QLC twice daily for 5 days [45]. The positive control group's treatment was carried out on the skin of the epidermis to the endodermis. Then, treatment was carried out twice a day for 5 days using an external drug in the form of a gel, The name is Bioplacenton Gel Tube containing 10% placenta extract and 0.5% neomycin produced by Kalbe Farma—Indonesia [46].

### 2.6. Statistical Analysis

Statistical analysis was conducted using Statistical Package for the Social Sciences (SPSS) IBM SPSS Statistics 27.0 software from IBM Corp. (New York, United States). Data on the incidence of proliferation between the trial group and control (positive and negative control) were calculated according to 5 days posttreatment to produce parametric data. Furthermore, a homogeneity test was conducted between data, and a comparison test was conducted. The observed values of epithelialization stimulation at levels 1 to 4 in the trial group during the treatment period were compared with the positive and negative control groups using independent *t*-test analysis with a significance level of 0.05. Furthermore, the determination of 50% endpoints was conducted using the probit analysis technique based on the trial group's observations. The value above the 50% endpoint was analyzed and referred to the probit test calculation.

## 3. Results

### 3.1. Isolation of QLC Using Preparative HPLC

**Figure 3:**
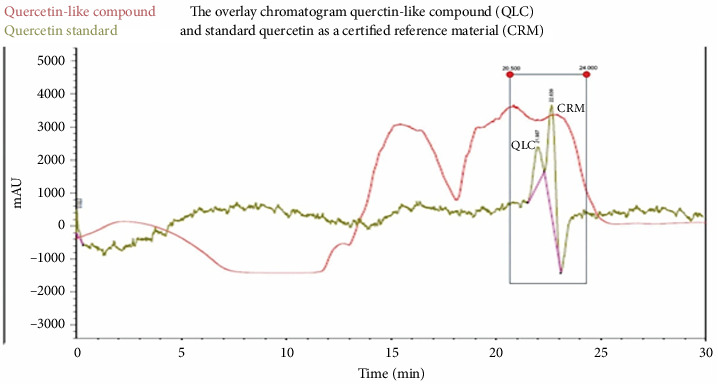
Chromatograms of quercetin-like compounds (QLC) at 10 μg/mL (red) and of quercetin standard at 10 μg/mL (magenta) at retention times of 21.987 and 22.639 min. Compounds were analyzed using preparative high-performance liquid chromatography 1620 Infinity II Agilent with diode array detector at 250, 370, and 375.4 nm in water:methanol mobile phase (40:60) using a C_18_ reverse-phase and flow rate of 0.5 mL/min with a stop time at 30 min. QLCs were collected at a retention time from 20.5 to 24 min as shown in the rectangular region.

The maceration of 3 kg of BD leaves produced 25 g of polyphenolic compounds. After testing the flavonoid content via the Willstatter technique, it was shown to contain flavonoids with a dark red color. Subsequently, the 25 g of polyphenol macerate was used for preparative HPLC to obtain 20.4 g QLC. The QLC sample isolates were eluted as a greenish-brown eluent. The QLC component was present in a yellow-colored methanol-water solution, which dried to a solid with a butter-like consistency. The analyte compounds isolated via preparative HPLC are shown in the chromatogram in Figure 3. The partition-adsorption process time was between 15 and 23 min, while the quercetin standard retention times peaked at 21.987 min and 22.639 min. Fractions were collected before the second standard peak and after the second raw sample peak for approximately 2 min [21, 22, 47]. Thus, QLC isolates were isolated between 15.5 and 24 min, as shown in Figure 3. The maceration of 3 kg of BD leaves produced 25 g of polyphenolic compounds. After testing the flavonoid content via the Willstatter technique, it was shown to contain flavonoids with a dark red color. Subsequently, the 25 g of polyphenol macerate was used for preparative HPLC to obtain 20.4 g QLC. The QLC sample isolates were eluted as a greenish-brown eluent. The QLC component was present in a yellow-colored methanol-water solution, which dried to a solid with a butter-like consistency. The analyte compounds isolated via preparative HPLC are shown in the chromatogram in Figure 3. The partition-adsorption process time was between 15 and 23 min, while the quercetin standard retention times peaked at 21.987 min and 22.639 min (Figure 3).

### 3.2. Identification Isolate QLC Using LC-ESI-MS

**Figure 4:**
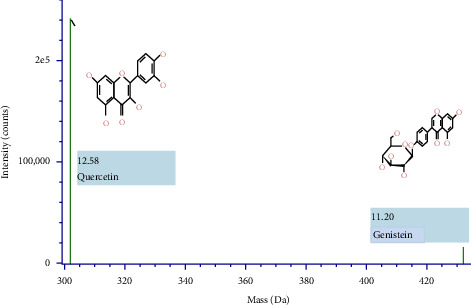
The molecule mass of quercetin at an intensity of approximately 2 × 10^4^ and genistein at approximately 24 × 10^4^ in QLC was observed using Q-TOF LC-ESI-MS type ultraperformance liquid chromatography-mass spectrometry and is controlled by the UNIFI software program (Waters Corp.).

Isolates were identified via the retractive technique of the adsorption-partition section in the region of the quercetin standard. The results of the molecular mass analysis are shown in Figure 4. The detailed composition isolates of QLC 4 show that isolate samples contained bioactive-like quercetin (C_15_H_10_0_7_) with a retention time of 12.57 min, 16 total fragments, isotope matches m/z RMS at 2.56 μg/mL, isotope match intensity of RMS% 3.20, response 272,394, and adduct at ^+^H. In contrast, bioactive-like genistein (C_21_H_20_O_10_) had a retention time of 11.20 min, 38 total fragments, isotope match m/z RMS at 2.43 μg/mL, isotope match intensity of RMS% 3.66, response 15,912, and adduct at ^+^H. The mass error of the test in bioactive-like quercetin was 2.5 μg/mL, whereas the mass error of the secondary metabolite genistein-like compound test was 2.1 μg/mL.

### 3.3. Identification of QLC Using FT-IR

**Figure 5:**
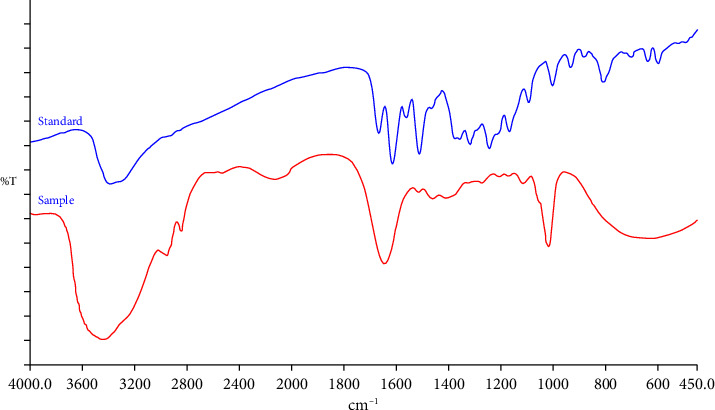
Spectra of Fourier transform infrared (FT-IR) spectroscopy for the quercetin-like compound sample (red line) and quercetin standard (blue line). FT-IR from PerkinElmer Spectrum One Models PN 09934358 by the potassium bromide pellet method with a parameter range of 4000–400 cm^−1^, duration scan of 20 s, and resolution of 4 cm^−1^.

The FT-IR analysis of QLC sample molecules is shown in Figure 5 from 450 to 4000 cm^−1^. Further analysis of the FT-IR examination at the specific region is shown in Table 3.

### 3.4. Standardization of Isolate QLC

The qualitative control for isolates of analytes using a fourth method stage is explained in Table 4.

### 3.5. Analysis of Particle Profile Using SEM

SEM photographs show that the morphology of QLC particles is generally rectangular with stacked shapes. The number of particles has a pointed tip, and the particle body has a variety of shapes ranging from cubes to rectangles. The particle thickness is between 0.01 and 0.1 μm. The formation of QLC particles with pointed ends is expected to attach to the fat-cell body. The surface of the particle body has horizontal grooves shaped like narrow gutters. The results of the surface area analysis using ImageJ and Origin software showed that all the surface areas of the particles that could be collected were normally distributed so that the proportion of particles with small areas was more dominant than that of particles with large areas. The part is lipophilic because of the extraction of polar organic solvents such as methanol-water. Thus, it will be able to penetrate body cells that are covered by fat. QLC particles are stable, provided they are not in organic solvents. When in organic solvents, QLC particles are unstable but able to bind other atoms from the external. The substances of QLC potentially produce compounds such as thromboembolic compounds for blood vessels that were obstructed by cholesterol fat. The grooved, gutter-like surface of QLC particles is thought to be the result of the HPLC-preparative column separation process. The image will be more deeply grooved if the diameter of the column is smaller than that of the column used [52, 53].

### 3.6. Topical Application of a Gel Containing QLC

**Figure 6:**
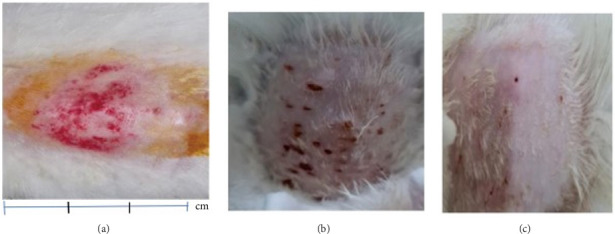
The development of epithelialization from level 1 to level 4 is shown in (a)–(c). (a) The injury site at the beginning of the test period (level 1). (b) The injury site after 2 days of the topical gel application period (level 2). (c) The injury site 5 days after gel application (level 4).

The proliferation effect, resulting from the application of topical gels containing QLC over 5 days on the scarred rabbit skin, significantly differed between the trial and control groups. As an answer to one of the hypotheses, the ability of epithelial proliferation in the trial group was real (*p* < 0.05; Table 5). Thus, topical gel therapy containing QLC from BD leaf extract stimulated the epithelialization of dermis cells in rabbits in the trial group. The probit analysis showed that an epithelialization of 25%, 50%, and 75% of the in vivo study endpoint was found at a QLC concentration of 4.644%, 5.185%, and 5.790%, respectively [54]. Example images of the application of QLC-topical gel on the proliferation of rabbit epidermis are shown in Figure 6. The scarred rabbit skin is shown in Figure 6(a) before applying the QLC-topical gels.  Figure 6(b) shows the injury site during the epithelial proliferation trial period (level 3), and Figure 6(c) shows the injury site at the end of the application period (level 4). The epithelialization test showed that the average control group would reach level 4 after 9 days, following 5 days of topical gel application. Thus, the epithelial proliferation period of the negative control group was 2 weeks. In the trial group, the epidermis 5 days after gel application showed that the skin epithelium proliferated quickly. The hairs grew faster and covered the postinjury area at the beginning of the study. So that 2 weeks after application of the gel, there will be no visible traces of skin wounds as when the skin wounds were first made.

## 4. Discussion

### 4.1. Analysis of Secondary Metabolite QLC

Multilevel percolation of 3 kg of BD leaves and pulverization produced approximately 25 g of macerated QLC with a final yield of 20.4 g of QLC. This amount was sufficient for use in identifying QLC and preparing QLC-topical gels, provided that the topical gel product does not exceed 65 g. The results of this study are, in principle, almost equivalent to other studies of plant extracts, which will obtain secondary metabolites around up to 1% of the crude extract [55, 56]. The difference with previous studies is that the secondary metabolite withdrawal technique uses a preparative HPLC device in this study, so it has a relatively higher purity. Since the number of isolates obtained by QLC was not excessive, for the limited number of QLC isolated, it would be preferable to collect BD leaves derived from the artificial cultivation of their host plant, *Lansium domesticum*. With the high use of BD leaves, efforts should be made to cultivate BD both naturally and artificially. The development of mistletoe can be encouraged in countries where the climate is very favorable for its development, as shown in Table 1. Mistletoes from bamboo plants and tea trees have been successfully cultivated through their host plants, indicating that BD plants can also be cultivated solely to develop new medicinal materials of plant origin [17, 21, 22]. A yellow QLC isolate solution was obtained, and the Willstatter test results indicated that the secondary metabolites obtained were similar to the quercetin standard used. QLC was identified in the flavonol component of the BD leaf extract. Therefore, the amount that can be isolated depends on the quantity of flavanols that can be extracted. The method used in this research extracts BD QLC, which contains many polyphenols in the form of salts with strongly electronegative atomic groups. This form of salt can easily infiltrate cells and stimulate new cell growth. This principle has been applied to pancreatic cells to encourage available cells to sustain insulin levels in the body, thus serving as a secondary metabolite for treating diabetes [48].

### 4.2. Analysis of QLC Isolated Using Preparative HPLC and LC-ESI MS

In the preparative HPLC, a shift of 1-2 min to the left and right of the peak of the standard chromatogram is the tolerance limit for isolating QLC (Figure 3). This is because of several reasons, one of which is that the polyphenol standards from plants generally have more than one peak. These peak fractions show that obtaining quercetin standards of high purity is difficult. The region where isolates were collected between retention times of 20.50 and 24.00 min is expected to contain a range of quercetin elements, with the collection of colored secondary metabolites believed to be QLC. In Figure 3, the magenta-colored standard chromatogram shows that no other peaks were present besides these two peaks. Thus, the collection of the peaks in this region ensures that the secondary metabolites isolated contain the core components of the two quercetin standard peaks [49]. The separation of natural materials of plant origin generally cannot be obtained with high purity. This is because each preparation depends on the differing quality of the nutrition of the plant and the ability of individual plants to perform the required metabolism [50]. Therefore, the quercetin standard also had two peaks, and this would certainly occur in the isolation results of QLC. Several researchers recommend that collections should occur at a maximum of 2-3 min before and after the isolation region of the standard used [51, 52]. By shifting the collection before and after this period, the QLC will be obtained with a purity of approximately 86%–90%. Figure 4 shows that the isolated QLC contains quercetin, also known as pentahydroxy flavone, which has five hydroxy groups at the 3-, 3′-, 4′-, 5-, and 7- positions. The QLC isolate also contained 7-hydroxyflavonol, which can conjugate with pentahydroxy flavone to form an acidic compound called quercetin-7-oleate. The QLC also contained genistein, also known as phytoestrogenic isoflavone, which can protect and stimulate the growth of eukaryotic cells and has benefits as a strong antioxidant. The QLC also contained 7-hydroxy-1-methoxy-2-methoxyxanthone and pelargonidin 3-glucoside [53]. These two compounds are strongly electronegative with negative ionic charges. Like quercetin, they have beneficial effects on internal eukaryotic cells, including cells in the Langerhans islets of the pancreas, and are useful as an antidiabetic treatment [54, 55]. The combination of genistein with quercetin-7-oleate and other elements in one derivative, such as morin, can be beneficial to eukaryotic cell life. The presence of secondary metabolites in QLC produces an acidic nature that is rich in hydroxyl groups and, therefore, will degrade the structure of viral RNA or bacterial DNA. Furthermore, this phenomenon is useful for new plant antibiotics. The hydroxyl group binds to polyphenol compounds in QLC, which will be useful as a stimulant for eukaryotic cell growth [56, 57].

### 4.3. FT-IR Analysis of QLC

The FT-IR analysis showed a peak at 632.38 cm^−1^, indicating the presence of carbon bonds with halogen atoms. The molecule was expected to be present in QLC and was closely related to strong electronegative groups such as iodine and bromine atoms [58]. The peak had a % *T* value that was not too strong, whereas the peak at 1018.19 cm^−1^ had a strong intensity and was CO−O−CO, a stretching anhydride as described in Table 3. This structure is also present in raw quercetin and is part of the main molecule of 7-hydroxy-flavonol [59, 60]. This structure also has a strong ability to stimulate the cell after penetrating the lipid-bilayer wall of the cell, which is electron-driving. Peaks were also present at 1271.84, 1205.51, 1169.27, and 1113.89 cm^−1^ but with less intensity, although they are included in the fingerprint region, indicating C−O stretching tertiary alcohol and aliphatic ether. These two elements are present in the standard quercetin and help stimulate the cell after binding to the component of the electron-propelling group of the lipid-bilayer cell surface. The peaks at 2125.07 and 2528.07 cm^−1^ with a lower % *T* are S−H stretching thiol while the peak at 2125.07 cm^−1^ with a low intensity was C ≡ C stretching monosubstituted alkyne. This bond is also found in the raw quercetin and can stabilize the molecular bonds incorporated in QLC. The peak at 1642.78 cm^−1^ outside the fingerprint region with a strong % *T* indicates that the molecule is a C=C stretching monosubstituted alkene. This peak was followed by less intense peaks at 1513.29, 1458.69, and 1408.54 cm^−1^. These bonds are also present in the quercetin standard with the difference that these elements have a stronger intensity in the quercetin standard than in the QLC. The bonds represented by these peaks are the N−O stretching nitro compound and C−H bending alkane methylene group, which have similar benefits as eukaryotic cell stimulants. The peak at 3436.98 cm^−1^ with a strong intensity is a −C− atom bound to H_2_O, while peaks at 2956.14 and 2843.69 cm^−1^ with less intensity represent C−H stretching alkane, which has been identified as a cell stimulant. The bond is also found in the standard quercetin. The strong electronegative groups that bind to the main 7-hydroxy-flavonol group or other polyphenols or even halogen elements are beneficial for direct interaction with the surface of eukaryotic cells so that the internal pathways of eukaryotic cells can directly respond with cellular defense [61–63].

### 4.4. Standardization of Isolates QLC

In the standardization of QLC isolates in Table 4, the effort showed that the separation analysis technique to obtain flavonol using the ALCl_3_ and three tube method was very accurate. The average of the results of the flavonoid content re-test using ALCl_3_ produces a chelation reaction, which reflected the complex bond between AlCl_3_ with keto (−CO−) and hydroxyl (−OH), resulting in a bluish color. The other method showed the same process as a chelation reaction on a green color of QLC isolates when the Zn powder was added to the tube of C. *R*_*f*_ analysis of QLC compared to the standard showed that the fate of adsorbed stationary phase (silica gel) analytes only has a similarity of 86.25%–97.14%. Thus, the polarity properties of the QLC and the standard are almost the same. Several studies on *R*_*f*_ values for quercetin compounds were obtained at 0.6 to 0.7 but depended on the composition of the mobile phase-stationary phase. The more acidic the mobile phase, the more analytes that contain QLC will be absorbed, especially keto and hydroxyl elements [64].

So, when the QLC isolate was re-tested against the adsorption-partition method chromatographically, it resulted in the finding of an analyte peak at a retention time during the 21–22 min. Sometimes, there were two peak areas in the region of retention time 8 min and 14 min, this was also found in the standard. This condition was possible considering the quercetin as a polyphenol structure obtained from plant material. However, the 3 peaks in the chromatogram were most stable in the 21–22 min region. So, the quantity of the presence of QLC as a second metabolite proliferant agent was obtained at the peak with a retention time of 21–22 min [65].

Analysis of the similarity of functional groups in the infrared fingerprint region shows that the intensity (%*T*) in the wave number (cm^−1^) region of 952.84 and 958.62 can be used as a standardization reference. This is because the shift in the peak of the infrared spectrum is not too far away, and the % *T* strength closely resembles [66, 67]. A review of the total quercetin content of the QLC isolates showed that the average quercetin content was between 60% and 61%. These results are similar to the findings of average quercetin content in other plants [68, 69].

### 4.5. Analysis of QLC Particles Used in a Topical Gel

**Figure 7:**
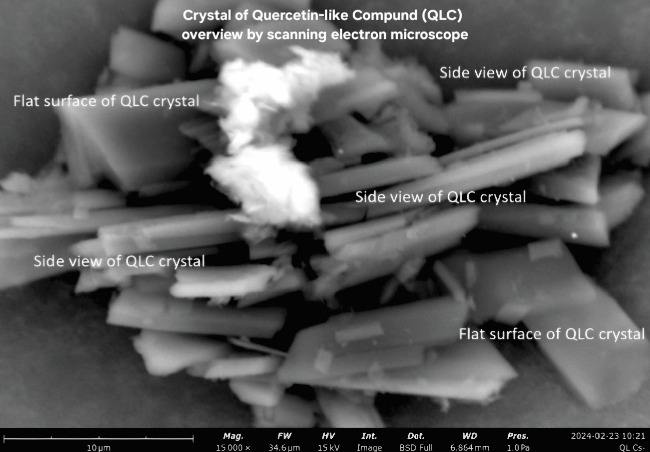
Crystal of QLC compounds at 15,000x magnification. Phenom ProX desktop scanning electron microscope with energy-dispersive X-ray diffraction adjusted field of works at 34.6 μm, high voltage 15 kv, detector using a full backscattered detector, working distance at 6.846 mm, and pressure at 1.0 Pa.

**Figure 8:**
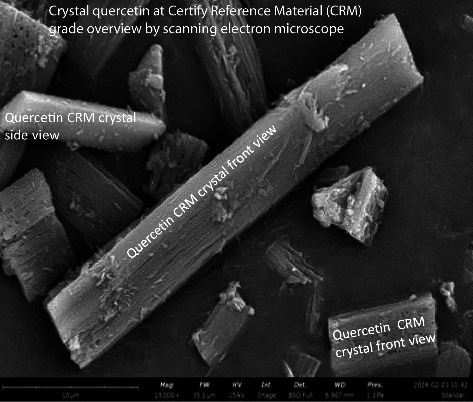
Crystal quercetin CRM grade at 15.000x magnification. Phenom ProX Desktop scanning electron microscope with energy-dispersive X-ray diffraction adjusted field of works at 35.1 μm, high voltage 15 kv, detector using a full backscattered detector, working distance at 6.846 mm, and pressure of 1.0 Pa.

The particle size of QLC is relatively small, with an average size of 7–12 μm, and therefore has a larger particle surface area compared to the standard of quercetin with particle sizes in the range of 15–22 μm (Figures 7 and 8). Several studies have isolated quercetin with an average particle size that is generally < 10 μm (Table 6). The isolation of quercetin in medicinal plants is straightforward, even if the amount obtained is small and can be used to produce intravenous injection dosage forms.

As the QLC particle size is small, a QLC solvent such as DMSO is not required. Note that DMSO solvent with a solubility ratio of 1:45 to 1:50 will disrupt the stability of the topical gel formulation. The amount of QLC obtained was limited, and the topical gel formulation could, therefore, only be produced up to 65 g, which decreased the stability of the topical gel formula. To avoid this, the QLC particles were dispersed using a homogenizer. Consequently, the distribution of particles into the topical gel base will be more proportional. The large surface area of the particles that will later interact with the cells also increases the possibility of interaction.

This study determined the composition of topical gels to produce a stable formula, as shown in Table 2. This formula required carbomer 1.6%, triethanolamine 1%, Na-EDTA 0.15%, and propylene glycol 23.08% to create a stable topical gel base formula. Triethanolamine works as a surfactant to dynamize the surface tension of topical gel preparations. This activity causes the gel to soften [74]. Therefore, the provision of secondary metabolites 4.6%, 6%, 8%, and 10% further strengthened the stability of the topical gels. The formula uses demineralized water, as the water component generally contains minerals, so determining demineralized water as a solution was intended to obtain mineral-free topical gels. The new formula was highly suitable for rabbit epithelial skin, with a pH between 6.8 and 7.2. The topical gel base formed (a) did not irritate the skin epithelium, (b) adhered easily, and (c) did not fall off easily when attached to the attachment at any skin location. Adding QLC between 4.5% and 10% will not change the pH range of the topical gel base.

### 4.6. Analysis of Stimulated Epithelialization of Epidermis Cells by Topical Gels Containing QLC

The secondary metabolites of QLC in topical gels promoted epithelial proliferation due to epithelial sloughing, as shown in Figure 6(a) (level 1, Figure 2). The QLC in 4.5% and 5.185% concentrations stimulated an increase in the epithelialization of epidermal cells up to 50% of the endpoint of the trial group, leading to recovery of the injury. The recovery profile of epidermal cell exfoliation (Figure 6(b)) showed that the number of *petechiae* in the wound had begun to decrease and the swelling around the wound area had begun to disappear, as shown by the level 3 criteria. At level 3 (Figure 2), the effect of the compressive test on the wound has begun to lessen so that the experimental animal no longer feels pain. When the recovery condition reached 75% of the endpoint with an estimated concentration of 5.790% QLC, the profile of the wound area resembled that in Figure 6(c) or reached level 4 (Figure 2). This will stimulate fur growth where the rabbit skin epithelium is exfoliated. A wound recovery to 90% endpoint could be obtained at a concentration of 6.394% QLC, and 99% endpoint recovery will be obtained at a concentration of 7.586% QLC. As an answer to the last hypothesis, it has been proven that this concentration will give a full proliferation ability. At these concentrations, all wounds should yield a level 4 type. In the negative control group, only 2 of 17 animals exhibited a level 3, and 5 days after treatment, all animals were still in level 2 (Figure 2). This shows that recovery did not occur, and no QLC intervention was performed. Up to 2 weeks after the trial period, the wound area was still at level 2 in rabbits in the negative control group, although this was increasing toward level 3 (Figure 2). After 1 month of the trial period, all rabbits in the negative control group had entered the recovery period (level 4, Figure 2). This phenomenon shows that the role of secondary metabolites in the QLC-topical gel can encourage re-epithelialization of the epidermis (*p* < 0.05). The results of the proliferation analysis of each trial and control group can be followed in Table 5. QLC of BD leaf extract in the development of exploration for therapeutic purposes is known to have real pharmacological properties, namely, as an antiviral, especially those with envelopes such as the Newcastle disease virus. In addition, it acts as a blood sugar lowering agent in diabetic rats [21, 22]. The other research on QLC of the plant was explored as a repellant compound from mosquito bites [75].

Skin epithelial proliferation can be stimulated through direct skin growth stimulation. In pigs, it is known that there is a gene that controls epithelial proliferation and this gene indicates the presence of a protein known as Secreted Frizzled-related Protein 2 (SFRP2). Meanwhile, it was known that SFRP2 stimulation was also influenced by the control of a protein known as Wingless and Int-1 (Wnt). Wnt has a control pathway to transduce genes, namely, β-catenin, and was dominant in basal cells. In the skin, it will dominantly work in the endodermis in cases of proliferation activity [76, 77]. The role of QLC with a polyaromatic molecular structure that has high energy and does not easily undergo re-arrangement is thought to stimulate transduction from the Wnt/β-catenin pathway. The stimulatory element arises because QLCs are rich in hydroxyl groups and primary amines. These elements indirectly provide proton donors for Wnt/β-catenin stimulation, thus indirectly promoting proliferation (mechanosensitive principle). This phenomenon is similar to the stimulation of QLC by the existence of β-Langerhans cells in the pancreas. Therefore, cases of blood sugar reduction can occur after QLC administration [78]. In principle, the particle size of a secondary metabolite compound greatly affects its pharmacological performance. Table 6 shows the particle size of each medicinal plant that affects its therapeutic performance.

The limitation of using rabbits in this study is that the speed of cell proliferation depends on the ability of the rabbit to consume the available feed. Thus, if the control group consumes a better diet than the test group, epithelial proliferation may occur faster in the control group. The main principle in stimulating epithelialization is using the polyphenol and equilibrium element 7-hydroxy flavone, which also contains a 7-hydroxy isoflavone with additional hydroxy groups at positions 5′ and 4′. It was stimulating cell proliferation with antioxidant properties as referred to in https://figshare.com/articles/journal_contribution/_b_The_activity_of_stimulation_of_mistletoe_b_b_extract_b_b_of_b_b_i_Dendropthoe_pentandra_i_b_b_L_Miq_to_squamosa_epidermis_cells_of_b_b_rabbit_b_/25599876 [79]. If the principle was aimed at targeting eukaryotic cells, it would be like the phenomenon of the rabbit trial group and using a concentration range between 4.5% QLC to 7.586% in a topical gel base like this study. The results of this research can also be indirectly utilized for human skin [80]. Another additional research limitation is that the number of samples in rabbits is not too large. This is due to the limited use of experimental animals. Sixty rabbits were needed as both the trial groups and the positive control and negative control group. Especially for particles of QLC, the particles should be homogeneously dispersed so that the functional groups bound to the main structure of the secondary metabolite, as in the fingerprint infrared spectroscopy region, will be in direct contact. The role of small particle size for rapid absorption into the pores of the outer layer of eukaryotic cells through the penetration mechanism, makes QLC work faster to induce cell growth. This fact can be found when treatment for 5 days of topical use on the wound area will show the speed of recovery). This phenomenon will not be separated from the influence of the small particles obtained from the separation and preparation of the HPLC column preparation [81, 82]. The weakness of this research was also that it is still not applied to animals classified as livestock, wild animals, and pet animals. Thus, the results of this study are specific to rabbits. A review of potential applications for various animal species. The technique is recommended for animals with hairy skin, such as dogs and cats, but is not suitable for species with movement skin type, like cattle. As an additional recommendation, it is necessary to conduct further research on QLC topical gel testing models on different wound models with variability in formulation mixtures to increase penetration.

## 5. Conclusions

This study showed, as an answer to the research hypothesis, that QLC at 4.5% concentration in a topical gel with a new formulation can stimulate the epithelialization of the top layer of exfoliated squamous cells of rabbit skin. In the statistical test, it was verified that the samples tested between the trial group, positive control group, and negative control group showed homogeneous distribution. The probit analysis indicated that the 4.5% concentration of QLC was sufficient to stimulate the epithelialization of epidermis cells (*p* < 0.05). We also found that the average recovery of epithelialization in rabbit epidermis at subcutaneous wound depth lasted up to 1 month. The recovery with topical application of the QLC-topical gel to a 65%–85% endpoint was found at concentrations of 5.523%–6.143% QLC with a particle dispersion size of < 10 μm. As a recommendation for research, further analysis of recovery in other types of animals, such as dogs and cats, animals with not-fixed skin such as cattle, and sweaty skin such as horses, should be conducted. The recommendation is related to the formula that was used in this study. The results of this study can also be utilized for the field of dermatology in humans with a note that the epidermis area in humans is adjusted, and the most appropriate is the hair area. Furthermore, the contribution of the phytopharmaceutical field for topical therapeutic use requires a fairly high purity, considering the impurity of QLC will cause unfavorable pharmacological effects that can be partial antagonists or antagonists.

## Figures and Tables

**Table 1:** An overview of the countries where mistletoe medicinal plant species can be found.

No	Species of plant	Therapeutic action	Area of plant	References
1	Mexican plant *Ageratina pichinchensis*	Wound healing and proliferation of epithelia in skin	Latin America	[10]
2	*Lobostemon fruticosus*, *Scabiosa columbaria*,	Wound healing	Africa	[11]
3	*Brassica campestris*, *Saccharum officinarum*, *Emblica officinalis*, *Trachyspermum ammi*, *Asparagus adscendens*, *Musa paradisica*, *Oryza sativa, Curcuma longa, Azadirachta indica, Tinospora cordifolia*, and *Tamarindus indica*.	Wound healing, proliferation epitel	Asia and South Asia	[12]
4	*Remijia ferruginea*	Proliferation of epithelia and antioxidant	Latin America	[13]

**Table 2:** Formulation of topical gels containing QLC from leaves of *Dendrophthoe pentandra* L. Miq.

Composition of formula topical gels	Amount used (g)			
QLC (g)	2.92	3.90	5.20	6.50
Carbomer 940 (g)	1.60	1.60	1.60	1.60
Triethanol amine (g)	0.65	0.65	0.65	0.65
Sodium-ethylenediamine tetra acetic acid (g)	0.10	0.10	0.10	0.10
Propylene glycol (g)	15.00	15.0	15.0	15.0
Demineralized water (mL)	Up to 65.00	Up to 65	Up to 65	Up to 65

**Table 3:** The evaluation results of the FT-IR analysis of the quercetin-like compound (QLC) and quercetin standard as certified reference material (CRM) at 1200 cm^−1^ to 700 cm^−1^.

No	QLC			Standard CRM			References
	Wave number (Cm^−1^)	Intensity (% T)	Prediction chemical groups	Wave number (Cm^−1^)	Intensity (% T)	Prediction chemical groups found	
1	1205.51	63.55	Aromatic conjugation to amine	1167.40	52.90	Aromatic conjugation to amine	[48, 49]
2	1169.27	63.78	C–O stretching vibration, as in ethers	1093.55	58.81	C–O stretching vibration, as an alcohol or ether, esters	
3	1113.89	62.17	C–O; S=O; stretching vibration, as in esters or conjugation to sulfoxide	1002.22	62.22	C–O stretching vibration, as in esters, alcohol	
4	1018.19	49.48	CO-O-CO stretching as an anhydride, or C–O as an alcohol	931.82	66.04	C–O stretching vibration, as in alcohols	
				880.88	68.10	C-H, bending	
				810.29	63.04	C-H, bending	

**Table 4:** Stages of standardization steps for raffinates quercetin like compound (QLC) from *Dendrophthoe pentandra* L. Miq leaf extract.

Stage	Method and instrument	Result	Reference
Stage 1^st^	The test containing flavonol on method I using iron chloride (III) solution 1%, and method II using three tubes A, B, and C, then sample C produced a green color	Raffinates 25 g of isolates using the method I produced a bluish color. The C tube was produced in green color	[21, 22, 50, 51]
Stage 2^nd^	Similar adsorption-partition at retention factor (*R*_*f*_) value between the analyte and the standard quercetin using the adsorption-partition method with thin-layer chromatography as a stationary phase and 1-butanol: acetic acid: pure water-free mineral (1:1:1) as a mobile phase	The *R*_*f*_ samples at 0.68 to 0.69 The *R*_*f*_ standard at 0.70 to 0.80	
Stage 3^rd^	Test quality peak area and retention time using high-performance liquid chromatography preparation type using an analytical column and a preparative column Similarity of active branch of substance in fingerprint wavenumber (cm^−1^) between the isolate and the standard of quercetin at 1200-700 cm^−1^	The peak area was single at 21–22 min retention time. The wavenumber 952.84 cm^−1^ at (% T) intensity 99.77 of samples similarity to 958.62 cm^−1^ at (% T) intensity 88.02 of standard quercetin	
Stage 4^th^	QLC containing keto and hydroxyl both binding to aluminum chloride (ALCl_3_), produced a yellow color, then observed by Spectrophotometer Ultra Violet -Visible at 392 nm	70 mg of isolates QLC contained approximately 42.17 mg quercetin	

**Table 5:** Proliferation effect of QLC isolated from mistletoe *Dendrophthoe pentandra* L. Miq (BD) on scraped rabbit skin 3 × 3 cm^2^ on the back, after 5 days of topical gel application twice a day compared with the positive control group and negative control group.

	Replicate	Level effect on trial groups with serial concentration gel^(a)^				*p*	
		4.5%	6%	8%	10%	Level effect of positive control group by Bioplacenton^(b)^	Level effect of negative control group by basis of gel^(c)^
Amount of rabbit	1^st^	3	3	4	4	15 rabbits at level 3, 5 rabbits at level 4.	2 rabbits at level 3, 18 rabbits at level 2.
	2^nd^	3	4	4	4		
	3^rd^	3	4	4	4		
	4^th^	3	4	4	4		
	5^th^	4	4	4	4	< 0.05	< 0.05

*Note:* The superscript for a versus b and a versus c in the same row differs significantly (*p* < 0.05). Level 1 to level 4: there are scrapings with a healing size of 75%–100%, 50%–75%, 25%–50%, and 0%–25%, from 3 × 3 cm^2^ scrapings accompanied by the appearance of new epithelial cells and pain reduced to no pain.

**Table 6:** Particle size of isolates quercetin from plants.

Compounds	Particle size (μm)	References
Quercetin mixing with other compounds made micelles form	22 nm	[70]
Quercetin pure	34.81–16.28 μg/mg	[71]
Quercetin purified from curcumin	192–282 nm, 178–277 nm	[72]
Quercetin-nanoliposome	134.11 nm	[73]

## Data Availability

The data that support the findings of this study are available on Figshare (https://doi.org/10.6084/m9.figshare.25599876.v27).

## References

[1] Fayiah M., Fayiah M. S., Saccoh S., Kallo M. K., Izah S. C., Ogwu M. C., Akram M. (2023). Value of Herbal Medicine to Sustainable Development. *Herbal Medicine Phytochemistry, Reference Series in Phytochemistry*.

[2] Wang B., Xie K., Lee K. (2021). Veterinary Drug Residues in Animal-Derived Foods: Sample Preparation and Analytical Methods. *Foods*.

[3] Nabi F., Ahmed J., Tao W. (2023). An Updated Review on Efficiency of Penthorum Chinense Pursh in Traditional Uses, Toxicology, and Clinical Trials. *BioMed Research International*.

[4] Bártíková H., Podlipná R., Skálová L. (2016). Veterinary Drugs in the Environment and Their Toxicity to Plants. *Chemosphere*.

[5] Khalifa H. O., Shikoray L., Mohamed M. Y. I., Habib I., Matsumoto T. (2024). Veterinary Drug Residues in the Food Chain as an Emerging Public Health Threat: Sources, Analytical Methods, Health Impacts, and Preventive Measures. *Foods*.

[6] Theerawatanasirikul S., Lueangaramkul V., Thangthamniyom N., Chankeeree P., Semkum P., Lekcharoensuk P. (2022). Andrographolide and Deoxyandrographolide Inhibit Protease and IFN-Antagonist Activities of Foot-And-Mouth Disease Virus 3Cpro. *Animals*.

[7] Habib H. M., El-Gendi H., El-Fakharany E. M. (2023). Antioxidant, Anti-inflammatory, Antimicrobial, and Anticancer Activities of Pomegranate Juice Concentrate. *Nutrients*.

[8] Jia J., Duan H., Liu B., Ma Y., Ma Y., Cai X. (2023). Alfalfa Xeno-miR168b Target CPT1A to Regulate Milk Fat Synthesis in Bovine Mammary Epithelial Cells. *Metabolites*.

[9] Mukandiwa L., Eloff J. N., Naidoo V. (2012). Evaluation of Plant Species Used Traditionally to Treat Myiasis for Activity on the Survival and Development of *Lucilia cuprina* and *Chrysomya marginalis* (Diptera: Calliphoridae). *Veterinary Parasitology*.

[10] Romero-Cerecero O., Zamilpa-Álvarez A., Ramos-Mora A. (2011). Effect on the Wound Healing Process and In Vitro Cell Proliferation by the Medicinal Mexican Plant Ageratina Pichinchensis. *Planta Medica*.

[11] Mhlongo F., Cordero-Maldonado M. L., Crawford A. D. (2022). Evaluation of the Wound Healing Properties of South African Medicinal Plants Using Zebrafish and In Vitro Bioassays. *Journal of Ethnopharmacology*.

[12] Sharma R., Manhas R. K., Magotra R. (2012). Ethnoveterinary Remedies of Diseases Among Milk Yielding Animals in Kathua, Jammu and Kashmir, India. *Journal of Ethnopharmacology*.

[13] Sarandy M. M., Gusmão L. J., Purgato G. A. (2022). Hydroalcoholic Extract of Remijia Ferruginea Accelerates the Closure of Skin Wounds by Modulating Tissue Morphology and Antioxidant Profile: An In Vitro and In Vivo Study. *Journal of Ethnopharmacology*.

[14] Poongavanam V., Wieske L. H. E., Peintner S., Erdélyi M., Kihlberg J. (2023). Molecular Chameleons in Drug Discovery. *Nature Reviews Chemistry*.

[15] Selwal N., Rahayu F., Herwati A. (2023). Enhancing Secondary Metabolite Production in Plants: Exploring Traditional and Modern Strategies. *Journal of agriculture and food research*.

[16] Az-zahra F. R., Sari N. L. W., Saputry R. (2021). Review: Traditional Knowledge of the Dayak Tribes (Borneo) in the Use of Medicinal Plants. *Biodiversitas*.

[17] Lazuardi M. (2008). The In Vitro Assessment of Anti-proliferation Activity of Crude Diethyl Ether Extract of Dendrophthoe Species against to Myeloma Culture Cell. *Dental Journal*.

[18] Lazuardi M., Hermanto B. (2014). *Dendrophthoe Pentandra* Methanolic Leaf Extract Increases Progesterone Levels in Female Rats. *Universal medicinal*.

[19] Yang D., Wang T., Long M., Li P. (2020). Quercetin: Its Main Pharmacological Activity and Potential Application in Clinical Medicine. *Oxidative Medicine and Cellular Longevity*.

[20] Kong D., Wang L., Niu Y. (2023). Dendrophthoe Falcata (L.f.) Ettingsh. And Dendrophthoe Pentandra (L.) Miq.: A Review of Traditional Medical Uses Phytochemistry, Pharmacology, Toxicity, and Applications. *Frontiers in Pharmacology*.

[21] Mochamad L., Malarvili S., Jasmine K., Lim V. (2024). *In Vitro* Analysis of Quercetin-like Compounds from Mistletoe *Dendrophthoe pentandra (L.) Miq*., as a Potential Antiviral Agent for Newcastle Disease. *F1000Research*.

[22] Lazuardi M., Anjani Q. K., Budiatin A. S., Restiadi T. I. (2024). Efficacy of Quercetin-like Compounds from the Mistletoe Plant of *Dendrophthoe pentandra L. Miq*, as Oral Random Blood Sugar Lowering Treatment in Diabetic Rats. *Veterinary Quarterly*.

[23] Zulkefli N., Che Zahari C. N. M., Sayuti N. H. (2023). Flavonoids as Potential Wound-Healing Molecules: Emphasis on Pathways Perspective. *International Journal of Molecular Sciences*.

[24] Toopkanloo S. P., Tan T. B., Abas F., Alharthi F. A., Nehdi I. A., Tan C. P. (2020). Impact of Quercetin Encapsulation With Added Phytosterols on Bilayer Membrane and Photothermal-Alteration of Novel Mixed Soy Lecithin-Based Liposome. *Nanomaterials*.

[25] Lyu Y., Liu S., Gao S., Zhou J. (2020). Identification and Characterization of Three Flavonoid 3-O-Glycosyltransferases from Epimedium Koreanum Nakai. *Biochemical Engineering Journal*.

[26] Stan D., Tanase C., Avram M. (2021). Wound Healing Applications of Creams and “Smart” Hydrogels. *Experimental Dermatology*.

[27] Özbilgin S., Acıkara Ö B., Akkol E. K., Süntar I., Keleş H., İşcan G. S. (2018). *In Vivo* Wound-healing Activity of Euphorbia Characias Subsp. Wulfenii: Isolation and Quantification of Quercetin Glycosides as Bioactive Compounds. *Journal of Ethnopharmacology*.

[28] Dobrecky C. B., Lucangioli S. E., Wagner M. L. (2022). The Argentine Mistletoes Ligaria Cuneifolia (Ruiz & Pav.) Tiegh (Loranthaceae) and Phoradendron Liga (Gillies Ex Hook. & Arn.) Eichler (Santalaceae) Thirty Years of Research. *Chemistry and Biodiversity*.

[29] Nandi S., De U. K., Chowdhury S. (2011). Current Status of Contagious Ecthyma or Orf Disease in Goat and Sheep—A Global Perspective. *Small Ruminant Research*.

[30] Fang W. C., Lan C. E. (2023). The Epidermal Keratinocyte as a Therapeutic Target for Management of Diabetic Wounds. *International Journal of Molecular Sciences*.

[31] Tottoli E. M., Dorati R., Genta I., Chiesa E., Pisani S., Conti B. (2020). Skin Wound Healing Process and New Emerging Technologies for Skin Wound Care and Regeneration. *Pharmaceutics*.

[32] Folle C., Marqués A., Díaz-Garrido N. (2024). Gel-Dispersed Nanostructured Lipid Carriers Loading Thymol Designed for Dermal Pathologies. *International Journal of Nanomedicine*.

[33] Dhal S., Pal A., Gramza-Michalowska A. (2023). Formulation and Characterization of Emulgel-Based Jelly Candy: A Preliminary Study on Nutraceutical Delivery. *Gels*.

[34] Petaloti A. I., Makri S., Achilias D. S. (2024). Bioactive Edible Gel Films Based on Wheat Flour and Glucose for Food Packaging Applications. *Gels*.

[35] Dihal A. A., Woutersen R. A., Ommen B. V., Rietjens I. M., Stierum R. H. (2006). Modulatory Effects of Quercetin on Proliferation and Differentiation of the Human Colorectal Cell Line Caco-2. *Cancer Letters*.

[36] Kobylińska A., Kobylińska A. (2016). Exogenous Quercetin as a Proliferation Stimulator in Tobacco BY-2 Cells. *Journal of elementology*.

[37] Aliu Y. O., Ödegaard S. (1983). Paired-Ion Extraction and High-Performance Liquid Chromatographic Determination of Diminazen in Plasma. *Journal of Chromatography A*.

[38] Mamman M., McKeever D. J., Aliu Y. O., Peregrine A. S. (1996). Pharmacokinetics of Diminazene in Plasma and Lymph of Goats. *American Journal of Veterinary Research*.

[39] Lazuardi M., Suharjono S., Chien C. H. (2022). Toxicity Test of Flavonoid Compounds from the Leaves of *Dendrophthoe pentandra* (L.) Miq. Using *In Vitro* Culture Cell Models. *Veterinary World*.

[40] Bagci N., Bayindir Z. S., Inal O., Altanlar N., Yuksel N. (2020). Development and In Vitro Evaluation of Nifedipine Gel Formulations for Anorectal Applications. *Current Drug Delivery*.

[41] Ahmed S., Mahmood S., Danish Ansari M., Gull A., Sharma N., Sultana Y. (2021). Nanostructured Lipid Carrier to Overcome Stratum Corneum Barrier for the Delivery of Agomelatine in Rat Brain; Formula Optimization, Characterization and Brain Distribution Study. *International Journal of Pharmaceutics*.

[42] Teimouri A., Yeung P., Agu R. U. (2021). Stability of Compounded Topical Nifedipine in Cream, Gel, and Ointment Bases. *International Journal of Pharmaceutical Compounding*.

[43] Domínguez-Oliva A., Hernández-Ávalos I., Martínez-Burnes J., Olmos-Hernández A., Verduzco-Mendoza A., Mota-Rojas D. (2023). The Importance of Animal Models in Biomedical Research: Current Insights and Applications. *Animals*.

[44] Mony M. P., Harmon K. A., Hess R., Dorafshar A. H., Shafikhani S. H. (2023). An Updated Review of Hypertrophic Scarring. *Cells*.

[45] Lipsitch M., Tchetgen Tchetgen E., Cohen T. (2010). Negative Controls: a Tool for Detecting Confounding and Bias in Observational Studies. *Epidemiology*.

[46] Rambe P. S., Putra I. B., Yosi A. (2022). The Effect of Roselle Leaf (*Hibiscus* sabdariffa L.) Extract Gel on Wound Healing. *Journal of medicine and life*.

[47] Bhushan A., Rani D., Tabassum M. (2023). HPLC-PDA Method for Quantification of Bioactive Compounds in Crude Extract and Fractions of Aucklandia Costus Falc. And Cytotoxicity Studies against Cancer Cells. *Molecules*.

[48] De Toledo T. A., Da Silva L. E., Botelho T. C. (2012). Characterization of Flavonoid 3-Methoxyquercetin Performed by FT-IR and FT-Raman Spectroscopies and DFT Calculations. *Journal of Molecular Structure*.

[49] Wulandari L., Retnaningtyas Y., Nuri L. H., Lukman H. (2016). Analysis of Flavonoid in Medicinal Plant Extract Using Infrared Spectroscopy and Chemometrics. *Journal of Analytical Methods in Chemistry*.

[50] Sankhalkar S., Vernekar V. (2016). Quantitative and Qualitative Analysis of Phenolic and Flavonoid Content in Moringa Oleifera Lam and Ocimum Tenuiflorum L. *Pharmacognosy Research*.

[51] Billowria K., Ali R., Rangra N. K. (2024). Bioactive Flavonoids: A Comprehensive Review on Pharmacokinetics and Analytical Aspects. *Critical Reviews in Analytical Chemistry*.

[52] Lazuardi M., Suharjomo S., Chien C. H. (2022). Encapsulation of Progesterone-like Compounds in 10% Liposome Increases Their Concentration in Rats Administered an Injectable Dosage Form of These Compounds. *Kafkas Universitesi Veteriner Fakultesi Dergisi*.

[53] Kant V., Jangir B. L., Nigam A., Kumar V., Sharma S. (2017). Dose Regulated Cutaneous Wound Healing Potential of Quercetin in Male Rats. *Wound Medicine*.

[54] Gutiérrez-Venegas G., Torras-Ceballos A., Gómez-Mora J. A., Fernández-Rojas B. (2017). Luteolin, Quercetin, Genistein and Quercetagetin Inhibit the Effects of Lipopolysaccharide Obtained from Porphyromonas Gingivalis in H9c2 Cardiomyoblasts. *Cellular & Molecular Biology Letters*.

[55] Memon A. H., Hamil M. S., Laghari M. (2016). A Comparative Study of Conventional and Supercritical Fluid Extraction Methods for the Recovery of Secondary Metabolites from Syzygium Campanulatum Korth. *Journal of Zhejiang University: Science B.*.

[56] Luthra R., Roy A., Pandit S., Prasad R. (2021). Biotechnological Methods for the Production of Ginsenosides. *South African Journal of Botany*.

[57] Aghababaei F., Hadidi M. (2023). Recent Advances in Potential Health Benefits of Quercetin. *Pharmaceuticals*.

[58] Smith B. C. (2023). Halogenated Organic Compounds. *Spectroscopy*.

[59] Catauro M., Papale F., Bollino F. (2015). Silica/Quercetin Sol–Gel Hybrids as Antioxidant Dental Implant Materials. *Science and Technology of Advanced Materials*.

[60] Awang M. A., Nik Mat Daud N. N. N., Mohd Ismail N. I., Abdullah F. I., Benjamin M. A. Z. (2023). A Review of Dendrophthoe Pentandra (Mistletoe): Phytomorphology, Extraction Techniques, Phytochemicals, and Biological Activities. *Processes*.

[61] Dias M. C., Pinto D. C. G. A., Silva A. M. S. (2021). Plant Flavonoids: Chemical Characteristics and Biological Activity. *Molecules*.

[62] Robertson J. L. (2018). The Lipid Bilayer Membrane and its Protein Constituents. *Journal of General Physiology*.

[63] Drew D., Boudker O. (2024). Ion and Lipid Orchestration of Secondary Active Transport. *Nature*.

[64] Tessema F. B., Gonfa Y. H., Asfaw T. B. (2023). Targeted HPTLC Profile, Quantification of Flavonoids and Phenolic Acids, and Antimicrobial Activity of Dodonaea Angustifolia (L.f.) Leaves and Flowers. *Molecules*.

[65] Dhillon A., Thakkar A., Sardana S. (2022). Development and Validation of HPLC and Spectrophotometric Method for the Quantification of Quercetin in Calendula Flower Extract. *International journal of pharmaceutical quality assurance*.

[66] Tripathy D. R., Singha Roy A., Dasgupta S. (2011). Complex Formation of Rutin and Quercetin With Copper Alters the Mode of Inhibition of Ribonuclease A. *FEBS Letters*.

[67] Sg L., Sethi S., Kundu A. (2025). Extraction of Polyphenolic Compounds from Rose and Marigold, UPLC-ESI-QToF-MS/MS, FTIR Characterization and Assessment of Antioxidant Activity. *Journal of applied research on medicinal and aromatic plants*.

[68] Neelam S. K. (2016). Investigation and Content Estimation of Quercetin in Psidium Guajava Leaves Extract. *Indian Drugs*.

[69] Baqer S. H., Al-Younis Z. K., Al-Shawi S. G. (2024). Extracting Quercetin from Different Plant Sources, Purifying it Using Different Extraction Methods (Chemical, Physical, and Enzymatic), and Measuring its Antioxidant Activity. *Frontiers in Bioscience*.

[70] Patel H. S., Shaikh S. J., Ray D. (2022). Formulation, Solubilization, and In Vitro Characterization of Quercetin-Incorporated Mixed Micelles of PEO-PPO-PEO Block Copolymers. *Applied Biochemistry and Biotechnology*.

[71] Ortiz-Mendoza N., San Miguel-Chávez R., Martínez-Gordillo M. J., Basurto-Peña F. A., Palma-Tenango M., Aguirre-Hernández E. (2023). Variation in Terpenoid and Flavonoid Content in Different Samples of Salvia Semiatrata Collected from Oaxaca, Mexico, and its Effects on Antinociceptive Activity. *Metabolites*.

[72] Sah M. K., Gautam B., Pokhrel K. P., Ghani L., Bhattarai A. (2023). Quantification of the Quercetin Nanoemulsion Technique Using Various Parameters. *Molecules*.

[73] Liu X., Yu S., Lu X. (2024). Optimization of Preparation Conditions for Quercetin Nanoliposomes Using Response Surface Methodology and Evaluation of Their Stability. *ACS Omega*.

[74] Barnes T. M., Mijaljica D., Townley J. P., Spada F., Harrison I. P. (2021). Vehicles for Drug Delivery and Cosmetic Moisturizers: Review and Comparison. *Pharmaceutics*.

[75] Lo W. L., Mok K. L., Yu Pui Ming S. D. (2018). Which Insect Repellents Should We Choose? Implications from Results of Local Market Survey and Review of Current Guidelines. *Hong Kong Journal of Emergency Medicine*.

[76] Sakamoto M., Morimoto N., Inoie M. (2017). Cultured Human Epidermis Combined With Meshed Skin Autografts Accelerates Epithelialization and Granulation Tissue Formation in a Rat Model. *Annals of Plastic Surgery*.

[77] Ledwon J. K., Vaca E. E., Huang C. C. (2022). Langerhans Cells and SFRP2/Wnt/Beta‐Catenin Signalling Control Adaptation of Skin Epidermis to Mechanical Stretching. *Journal of Cellular and Molecular Medicine*.

[78] Nakano T., Sakamoto M., Katayama Y. (2023). Dried Human-Cultured Epidermis Accelerates Wound Healing in a Porcine Partial-Thickness Skin Defect Model. *Regenerative therapy*.

[79] Sychrová A., Škovranová G., Čulenová M., Bittner Fialová S. (2022). Prenylated Flavonoids in Topical Infections and Wound Healing. *Molecules*.

[80] Taiki C., Yuudai H. (2012). *Quercetin: Dietary Sources, Functions and Health Benefits (Nutrition and Diet Research Progress)*.

[81] Bareford L. M., Swaan P. W. (2007). Endocytic Mechanisms for Targeted Drug Delivery. *Advanced Drug Delivery Reviews*.

[82] Kamena F., Monnanda B., Makou D., Capone S., Patora‐Komisarska K., Seebach D. (2011). On the Mechanism of Eukaryotic Cell Penetration by α- and β-Oligoarginines: Targeting Infected Erythrocytes. *Chemistry and Biodiversity*.

